# Supplementary figures and images for: Interpreting the CTCF-mediated sequence grammar of genome folding with AkitaV2
Source: PLoS Comput Biol. 2025 Feb 4;21(2):e1012824. doi: 10.1371/journal.pcbi.1012824 (PMC11828424; doi:10.1371/journal.pcbi.1012824)

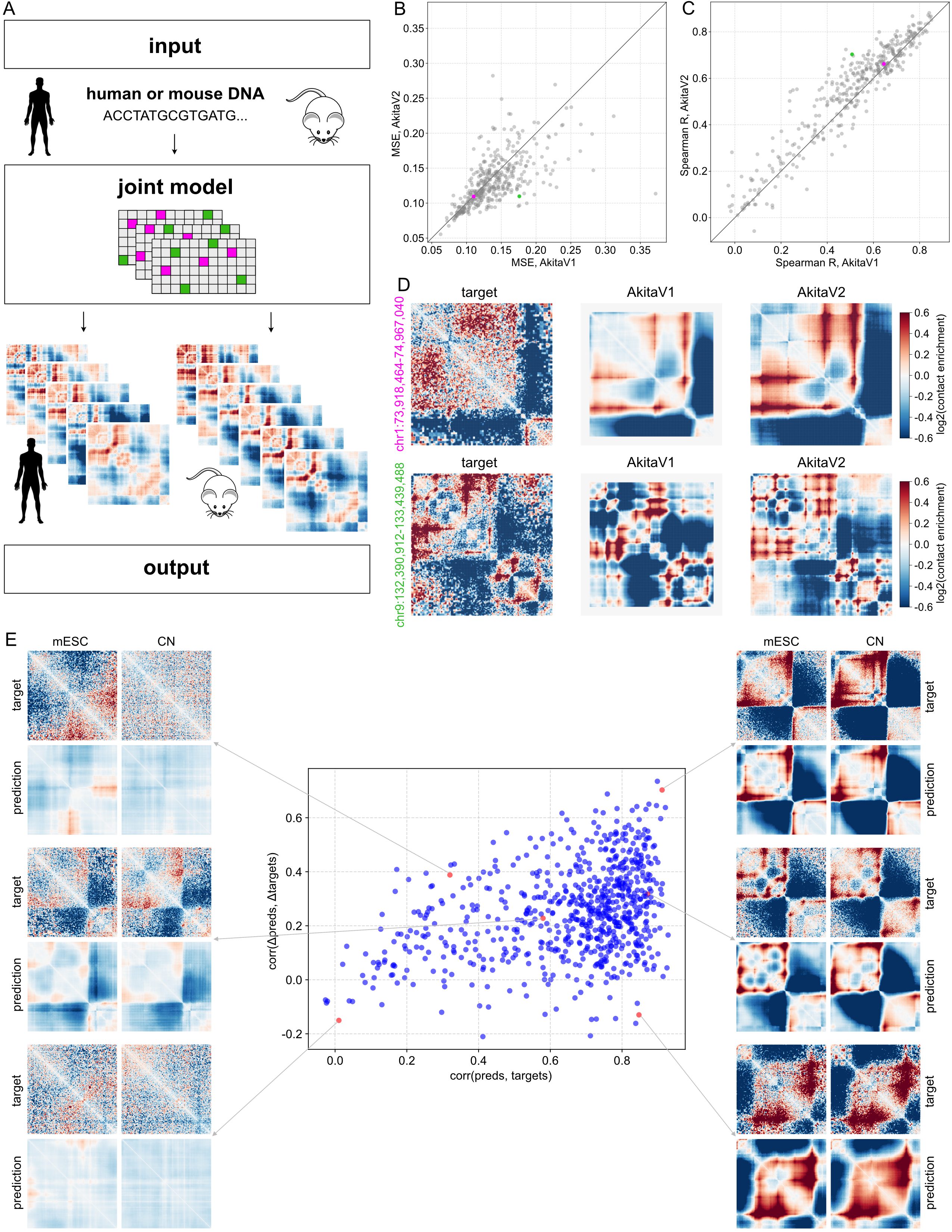

Supplement: S1 Fig — A) AkitaV2 architecture. This model inputs ~1.3 million base pairs of DNA to predict log(observed/expected) pairwise contact frequencies. The model employs a shared trunk and two distinct prediction heads: one for six mouse cell types and another for five human cell types. B) Scatterplot of MSE for AkitaV1 vs. the AkitaV2 human predictions for each genomic region in the overlap between AkitaV1 and AkitaV2 test sets, making use of how each AktiaV2 region was in the test set for one of the eight AkitaV2 models. We overlapped the test set from AkitaV1 (413 sequences) and that for all models from AkitaV2 (5841 sequences) using an inner join, yielding 400 regions with substantial overlap. We then selected the prediction for the AkitaV2 model that had this region in its test set. For a conservative comparison we cropped AkitaV2’s prediction to match the size of AkitaV1’s predictions, as AkitaV2 generates larger contact maps by design, though this slightly underestimates the increased performance for AkitaV2. AkitaV2 displays enhanced performance (0.131 vs. 0.139). Pink and green dots highlight two representative genomic regions: one where AkitaV2 prediction is comparable with AkitaV1, and one where the AkitaV2 prediction outperformed AkitaV1. Predicted maps for the two highlighted regions are shown below. C) Scatterplot of Spearman correlation coefficients for AkitaV1 vs AkitaV2 for regions in the same test set as in (B). Akita displayed improved Spearman R (0.59 vs. 0.56) and Pearson R (0.66 vs. 0.62) across the test set. Colored dots as in (B). D) Visual comparison of log(observed/expected) contact frequencies for a genomic window with minimal improvement. From left to right: the experimental target map, the prediction by AkitaV1, and the prediction by AkitaV2, all for the human HFF model output. E) Comparative analysis of AkitaV2 predictions for mESCs and cortical neurons (CN), both from Bonev et al., 2017 [34]. In the central scatterplot, each point represents correla [file pcbi.1012824.s002.tiff]

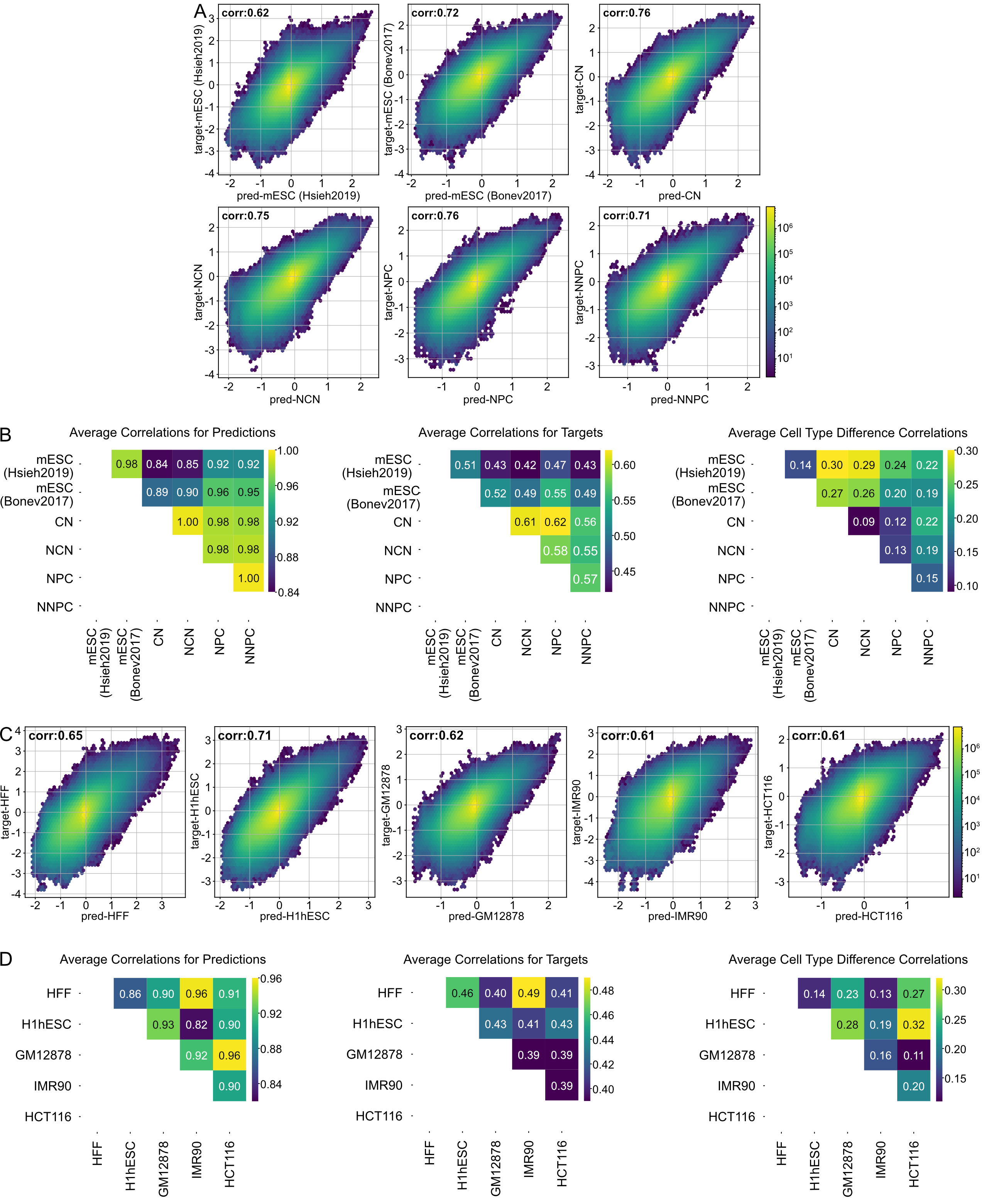

Supplement: S2 Fig — A) Predicted vs. experimental log(observed/expected) values for each bin pair across all regions in the test set for mouse model 0, shown separately for each target. The plot demonstrates a correlation between predictions and experimental data across cell types. Colors represent the log10 number of bin pairs for each set of predicted vs. experimental values, and Pearson R is provided as a measure of correlation. Cell types are abbreviated as: mouse embryonic stem cells (mESC), cortical neurons (CN), neocortex cortical neurons (NCN), neural progenitor cells (NPC), neocortex neural progenitor cells (NNPC). B) Across all regions in the mouse model 0 test set for different cell types, we observe the following: Left: model predictions are highly correlated between cell types (Pearson R(pred(i,j,c1), pred(i,j,c2)), where c1 and c2 denote cell types, and the correlation is computed across all genomic regions i and pixels j). Middle: experimentally assayed genome folding shows correlations between cell types, though these are weaker (Pearson R(targets(i,j,c1), targets(i,j,c2)). Right: predicted cell-type differences from our models show weak correlations with observed differences (Pearson R(pred(i,j,c1)—pred(i,j,c2), targets(i,j,c1)—targets(i,j,c2)). Note that the scales for Pearson R differ across the panels. C) As in A), for human model 0. D) As in B), for human model 0. (TIFF) [file pcbi.1012824.s003.tiff]

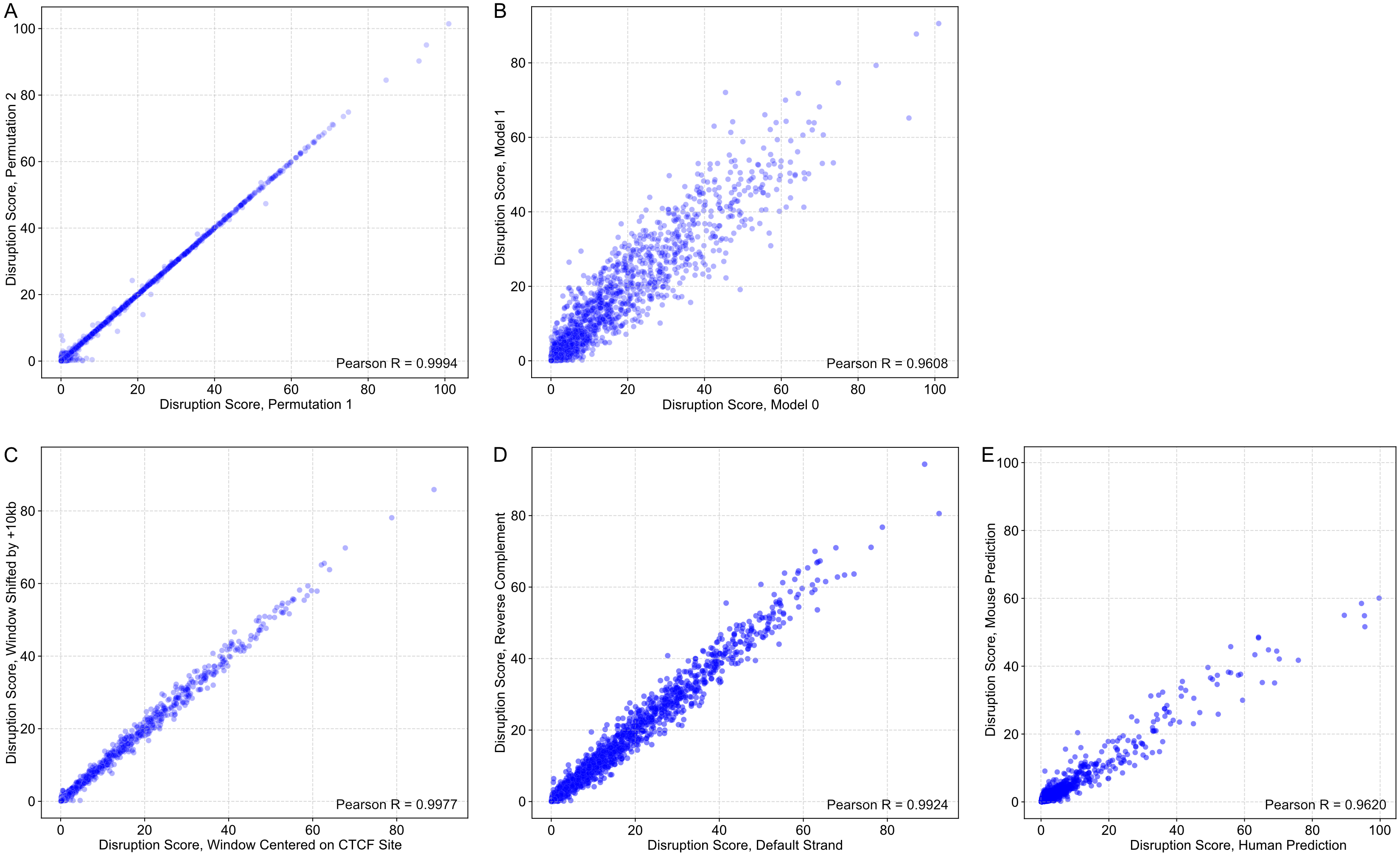

Supplement: S3 Fig — A) Disruption scores are highly correlated for random CTCF permutations. Scatterplot of disruption scores for n = 7,560 individual CTCF sites subjected to random permutations, where each point represents the predicted disruption score of an individual CTCF site. Disruption scores were computed twice (with model 0) for each CTCF-binding site overlapping a TAD boundary. B) Inter-model consistency in CTCF site disruption. Scatterplot of disruption scores for n = 7,560 individual CTCF sites compared between model 0 and model 1. Disruption scores are highly correlated across all pairs of models 0–7 (PearsonR > 0.955). C) Position of disrupted CTCF site relative to the prediction window. This plot explores the effect of shifting the predictive window by +10kb. We tested shifts of ±10kb, ±1kb, ±100bp, ±10bp, and ±1bp, comparing the scores for each shift with the scores from the centered permutation. All correlation coefficients between the disruption scores for shifted permutations and the centered permutation were consistently high, exceeding 0.997. D) Consistency in CTCF sites disruption across DNA strands. Disruption scores do not depend on the input sequence orientation. E) Inter-species consistency of CTCF site disruption. 18Mb of mouse chromosome 1 (ch1:3,653,632–21,776,376) was disrupted by permuting a sliding ~200bp window and disruption scores were calculated for either the human (hESC) or mouse (mESC) output. Scatterplot shows median disruption score across four models for each 200bp window for the human versus the mouse output (Pearson R > 0.96). (TIFF) [file pcbi.1012824.s004.tiff]

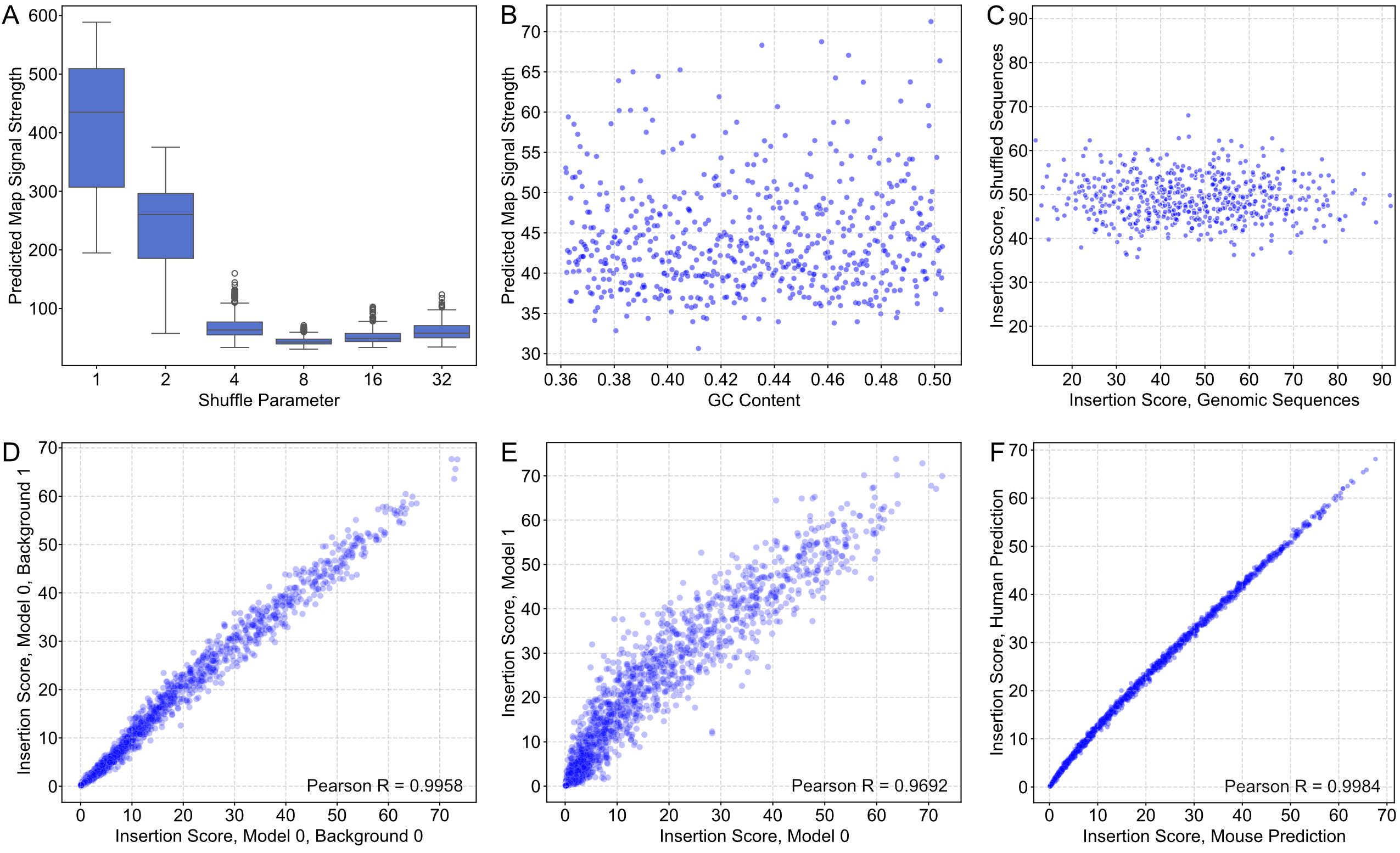

Supplement: S4 Fig — A) Boxplots constructed from predicted map signal strength scores of n = 590 shuffled genomic windows show DNA sequence shuffling impact on contact matrix strength, with predicted map signal strength for model 0 across shuffled sequences (1, 2, 4, 8, 16, 32 nucleotides). Lower scores denote weaker maps, with k = 8 shuffling resulting in the most neutral maps. B) Scatterplot of predicted map signal strength versus GC content for shuffled genomic sequences. Points represent scores from model 0 for n = 590 genomic windows shuffled using k = 8, and show no trend between GC content and SCD. C) Scatterplot comparing insertion scores for the insertion of a strong CTCF site into n = 590 genomic sequences, both original and shuffled once with k = 8. While shuffling does not alter the mean, it remarkably reduces the variance of the insertion scores. D) Scatterplot of virtual insertion score for background sequence 0 vs. background sequence 1 for model 0 across n = 7,560 CTCF sites. Virtual insertion scores are highly correlated across backgrounds (PearsonR > 0.94 for any pair of background sequences). E) Scatterplot of insertion scores between model 0 and model 1 for n = 7560 CTCF sites. Virtual insertion scores are highly correlated across pairs of models (PearsonR > 0.96). F) Scatterplot of insertion scores between mouse and human predictions for n = 7560 mouse CTCF sites inserted into shuffled mouse background sequences. Insertion scores are highly correlated (PearsonR > 0.99). (TIFF) [file pcbi.1012824.s005.tiff]

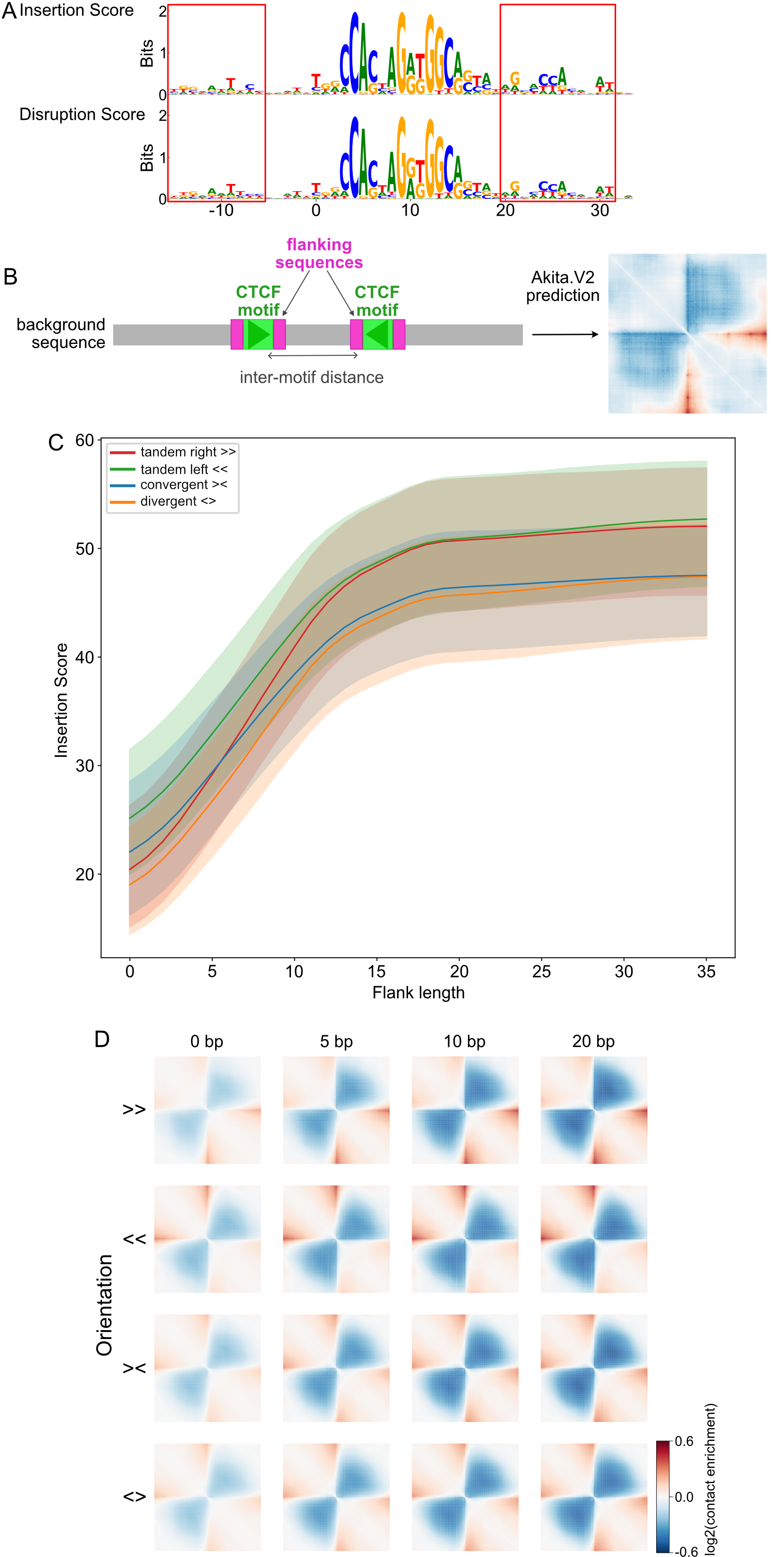

Supplement: S5 Fig — A) Sequence logos for the strongest 150 CTCF sites ranked by either insertion (top) or disruption (bottom) scores. Red boxes highlight weak sequence preferences upstream and downstream of the CTCF core motif. B) Illustration of a double CTCF site insertion. Two CTCF sites (green boxes) are virtually inserted symmetrically around a background sequence’s midpoint (gray rectangle) with constant 180bp spacing (as in [15]) with flanking regions (pink boxes). The impact is quantified by the squared contact difference between maps with and without inserted CTCF sites (insertion score). C) Insertion score versus flanking sequence length for insertions of tandem CTCF sites in four orientations (left, right, convergent, divergent). Grouping and shading as in Fig 6B. Insertions of tandem sites show a similar trend to single sites, and similar trends across all orientations. D) Predicted maps for double CTCF insertions with increasing flank length for different orientations. Inserted CTCF sequence extracted from chr7:37,357,852–37,357,871. The maps are arranged in a grid by flank length (columns) and site orientation (left, right, convergent, divergent) in rows, demonstrating similar strength impacts across orientations but slight asymmetry for sites inserted in tandem. (TIFF) [file pcbi.1012824.s006.tiff]

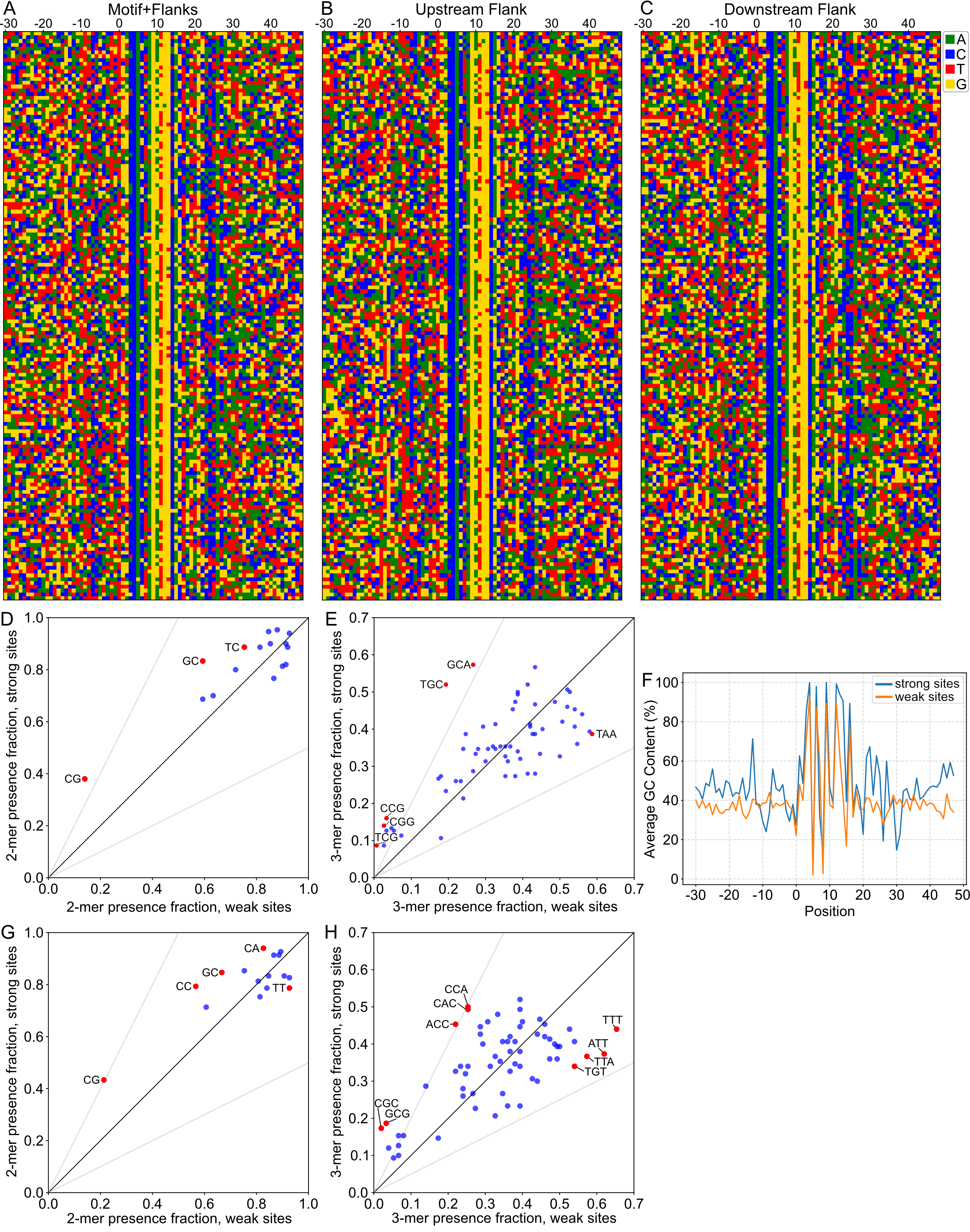

Supplement: S6 Fig — A) Heatmap of nucleotide composition around 150 strong CTCF sites (±30bp), with rows sorted according to the Hamming distance between their sequences. B) Same as panel A except rows are ordered by the Hamming distance between upstream flanking sequences. C) Same as panel A and B, but with rows arranged by the Hamming distance between downstream flanking sequences. D) Scatterplot showing the fraction of strong and weak upstream flanking sequences containing k-mers (k = 2). Strong and weak sequences are defined as the 150 CTCF sites with the highest and lowest insertion scores, respectively. Red dots indicate significantly enriched or depleted k-mers in strong versus weak flanking regions. Bootstrap sampling (n = 100,000) was used to generate distributions of differences in k-mer presence fractions. These distributions were used to assess significance (at 0.05 corrected by the number of k-mers tested) of k-mers enrichment (above zero) or depletion (below zero). Grey lines represent 2-fold up and down ratios. E) Same as panel D, but for k = 3. F) Average GC content (%) for 150 strong (blue) and 150 weak (orange) CTCF sites. G) Same as panel D, but for k-mer enrichment in downstream flanking regions. H) Same as panel E, but for k-mer enrichment in downstream flanking regions. (TIFF) [file pcbi.1012824.s007.tiff]

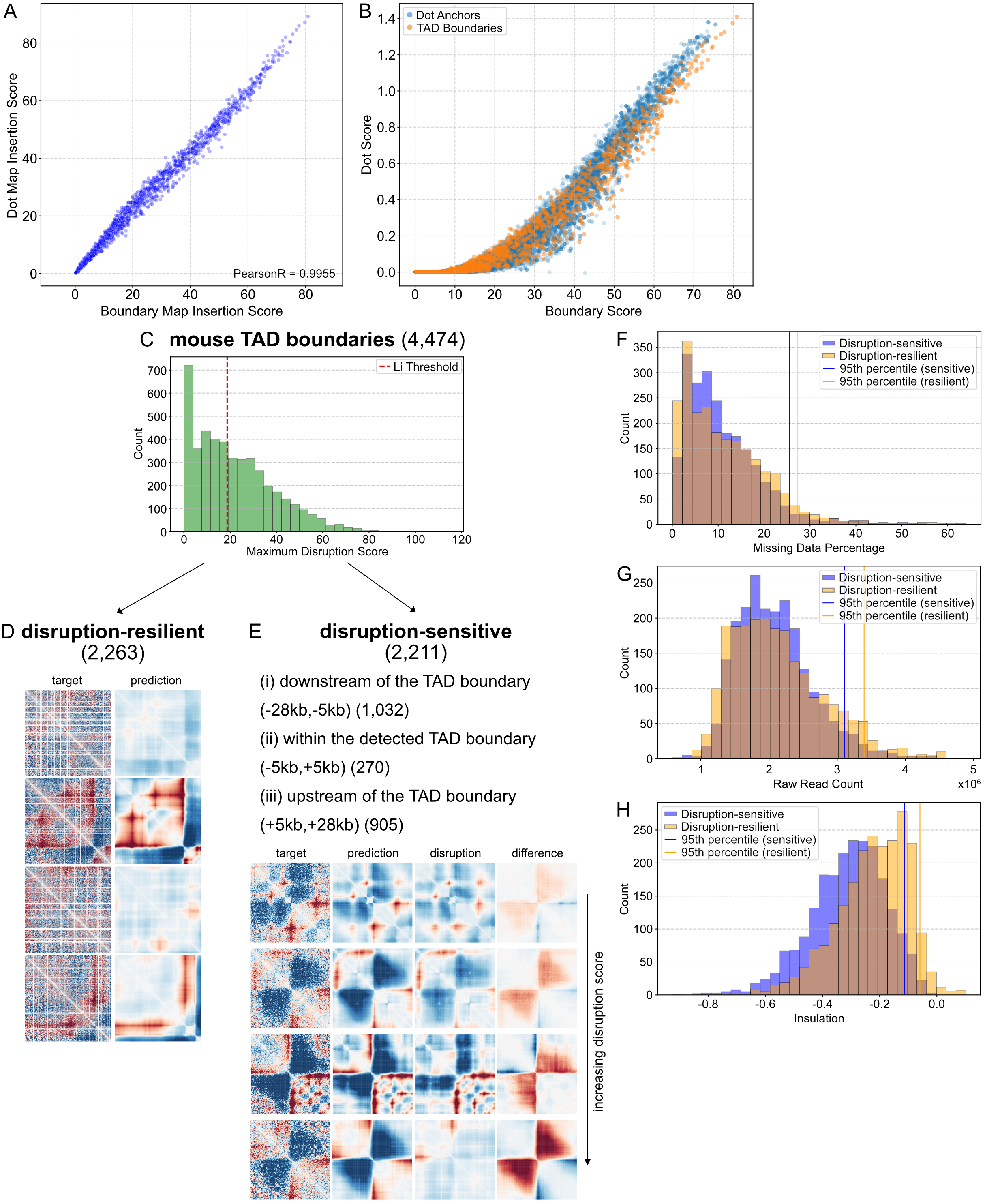

Supplement: S7 Fig — A) Scatterplot of insertion scores from boundary versus dot scenarios shows a high correlation (PearsonR > 0.99), indicating that the global metric of predicted map strength does not significantly vary with the insertion scenario. B) CTCF sites with differing genomic origins have similar dot and boundary strengths. Scatterplot shows dot versus boundary strengths for two sets of CTCF sites, either: overlapping TAD boundaries (orange dots, n = 1,500), or dot anchors (blue dots, n>36,900) called in experimental Hi-C maps. These sets of CTCF sites are disjoint. Uniform distribution across the plot shows all sites behave similarly in the experiment, regardless of their genomic origin. This suggests that CTCF’s role in chromatin architecture does not inherently differ between those overlapping with TAD boundaries and those at dot anchors. C) TAD disruption analysis in mouse embryonic stem cells (mESCs) at 10-kb resolution. The Akita model was used to evaluate the impact of permuting 2048-bp sequences within or near 4,474 TAD boundaries. The histogram depicts the distribution of maximum disruption scores per boundary, with the red dashed line representing the Li threshold. This threshold separates disruption-sensitive boundaries (high scores) from disruption-resilient boundaries (low scores). Of the disruption-sensitive boundaries, 751 overlapped with transcription start sites (TSSs), compared to 828 overlaps in the disruption-resilient group. D) Disruption-resilient boundaries (2,263 total) lacked evidence of TAD boundary disappearance and often displayed higher levels of missing data and less accurate predictions. E) Disruption-sensitive boundaries (2,211 total) were characterized by the disappearance of TAD boundaries in predicted Hi-C maps. F) Disruption-resilient boundaries were enriched for regions with missing bins. G) Disruption-resilient boundaries showed enrichment in areas with low sequencing coverage. H) Disruption-resilient boundaries corresponded to regions l [file pcbi.1012824.s008.tiff]
